# Evolution of polygenic traits under global *vs* local adaptation

**DOI:** 10.1093/genetics/iyab134

**Published:** 2022-01-04

**Authors:** Sam Yeaman

**Affiliations:** Department of Biological Sciences, University of Calgary, Calgary, AB T2N 1N4, Canada

**Keywords:** local adaptation, global adaptation, genetic architecture, selection, migration, recombination, genome scan

## Abstract

Observations about the number, frequency, effect size, and genomic distribution of alleles associated with complex traits must be interpreted in light of evolutionary process. These characteristics, which constitute a trait’s genetic architecture, can dramatically affect evolutionary outcomes in applications from agriculture to medicine, and can provide a window into how evolution works. Here, I review theoretical predictions about the evolution of genetic architecture under spatially homogeneous, global adaptation as compared with spatially heterogeneous, local adaptation. Due to the tension between divergent selection and migration, local adaptation can favor “concentrated” genetic architectures that are enriched for alleles of larger effect, clustered in a smaller number of genomic regions, relative to expectations under global adaptation. However, the evolution of such architectures may be limited by many factors, including the genotypic redundancy of the trait, mutation rate, and temporal variability of environment. I review the circumstances in which predictions differ for global *vs* local adaptation and discuss where progress can be made in testing hypotheses using data from natural populations and lab experiments. As the field of comparative population genomics expands in scope, differences in architecture among traits and species will provide insights into how evolution works, and such differences must be interpreted in light of which kind of selection has been operating.

## Introduction

The process of adaptation is central to evolution, and many fundamental questions are oriented toward understanding the nature of evolutionary potential and the factors that constrain it (Gould and Lewontin 1979). One way to understand the balance between potential and constraint in evolution is to study repeatability in adaptation—if we see the same gene(s) contributing in response to the same selection pressure, we can study why this happens. On the one hand, this can be seen as a clear expression of evolutionary potential: we might conclude that gene *x* contributes to *y* response in several different species because it is the best gene for the job. On the other hand, we may wonder why genes *a*, *b*, and *c* did not contribute to *y* response in any species, especially if they affect the same trait as gene *x*. By comparative study of the genetic architecture of adaptation, we can begin to understand the fundamental nature of evolutionary potential and constraint. However, if we are to make clear interpretations about any observed differences in architecture, it is critical to have clear predictions about how different kinds of selection shape it. My broad aim here is to review current data and analyses about the genetic basis of trait variation and adaptation and relate this to predictions about evolution under global *vs* local adaptation. I will pay particular attention to the importance of genotypic redundancy (*i.e*., multiple genotypes producing the same phenotype), as it has important impacts on model predictions and also is explicitly connected to understanding the concept of evolutionary constraint. However, as the connections between redundancy and constraint have been discussed previously (Yeaman *et al*., 2018), this study will focus on how redundancy affects predictions about global *vs* local adaptation.

## The nature of adaptive genetic variation: insights from genomics

Genome Wide Association Studies (GWAS) in humans and other organisms have often found that trait variation is driven mainly by alleles of small effect (Visscher *et al.* 2017; Sella and Barton 2019). Coupled with the observation that there is very little evidence for new beneficial mutations having swept rapidly through the human populations (*i.e.*, hard selective sweeps; Pritchard and Di Rienzo 2010), this has prompted extensive discussion about the genetic basis of complex traits and how adaptation works (Boyle *et al.* 2017; Wray *et al.* 2018; Sella and Barton 2019; Barghi *et al.* 2020). Classical population genetics describes adaptive evolution in terms of allele frequency changes at individual loci, which each experience selection. If individual loci experience strong selection, then large changes in allele frequency are expected during adaptation. By contrast, in the quantitative genetics paradigm, models assume that many alleles have small and approximately interchangeable effects on a trait, so that large changes in trait value can be achieved through small shifts in allele frequency across many loci. While complementary (Fisher 1930; Johnson and Barton 2005), the foundational assumptions of these models imply very different predictions about the expected genomic signature of adaptation: does it progress by a few big sweeps or many small shifts? (Höllinger *et al.*  2019). It is clear that some loci in humans experience strong individual selection, such as the textbook examples of alleles responsible for sickle cell anemia (Elguero *et al.* 2015) and lactase persistence (Tishkoff *et al.* 2007). However, the absence of a large number of selective sweep signatures and the preponderance of variants of small effect in GWAS suggest that much of the variation responsible for human traits may be difficult to detect (Manolio *et al.* 2009; Visscher *et al.* 2017).

Moving outside of a human-centric view of evolution, findings on the genetic basis of trait variation become a little more varied. Selective sweeps have been found in *Drosophila* (Vy *et al.* 2017), mice (Ilhe *et al.* 2006), and many other species (Huber *et al.* 2016; Booker *et al.* 2017; Stephan 2019). Estimates of the proportion of amino acid changing nucleotide substitutions that are fixed by selection tend to commonly find large values (Galtier 2016; Booker *et al.* 2017). This suggests that selection drives much of the long-term evolution in genome sequence, implying there are many mutations with *s*** **>** **1/N_e_ (*i.e.*, the threshold where selection becomes efficient relative to drift; Wright 1931; Crow and Kimura 1970; See Table 1 for definition of symbols). Some of the most celebrated examples of adaptation have revealed variants of large effect: beak size in the iconic Darwin’s finches is driven in part by a variant with a selection coefficient of *s*** **=** **0.59 (Lamichhaney *et al.* 2016), *Mc1r* seems to crop up almost every time someone studies color pattern in vertebrates (Manceau *et al.* 2010), and numerous loci of large effect have now been identified controlling a range of adaptive traits in threespine stickleback (*e.g*., Shapiro *et al.* 2004; Colosimo *et al.* 2005). On the other hand, it also seems clear that much adaptive variation is controlled by alleles of small effect (Rockman 2012), that adaptation from standing variation is a common mode of evolution (Hermisson and Pennings 2005; Stephan 2019), and that identifying all causal variants may be just as difficult in nonhuman organisms. There has been some debate about what can be accomplished in the search for the loci responsible for adaptation (Rockman 2012; Martin and Orgogozo 2013; Travisano and Shaw 2013; Lee *et al.* 2014), and to some extent the answer to this question must depend upon whether adaptation is driven by a few big sweeps or many small shifts.

As in most problems in biology, the true answer likely falls somewhere between these two extremes. Of course, while this facile answer is almost surely correct, it glosses over the importance of trends that seem to be found in nature. For example, it is interesting that many of the adaptive alleles of large effect that have been discovered to this point (reviewed in Martin and Orgogozo 2013; Rees *et al.* 2020) are responsible for driving local, rather than global adaptation (or are under some form of balancing selection). My aim in this review is to explore how our understanding of the genetic basis of trait variation is shaped by the context in which we study adaptation: whether the phenotype of a species evolves toward a single (global) optimum or a spatially varying (local) optimum. Differences between these two regimes in the way that selection interacts with drift and migration can result in some dramatic differences in the predicted outcomes of adaptation. By better understanding the differences in such predictions, we can be better prepared to interpret the differences we may see among the genetic architectures of adaptation, which will give clearer insights into how evolution works.

Global adaptation is a spatially explicit version of the standard conception of how evolution leads to the gradual refinement of a trait within a species, such as the evolution of opposable thumbs in ancestral humans, which presumably evolved because this was a beneficial trait in all environments they encountered. Global adaptation can be defined at the phenotypic level, where all populations of a species experience selection toward the same optimum, or at the allelic level, where a given allele has the highest average fitness across the range of the species and natural selection tends to favor its fixation throughout. In either case, global adaptation tends to behave approximately according to dynamics expected for a single population under directional selection, but with some modifications due to the effect of spatial structure.

By contrast, local adaptation occurs when an organism inhabits a heterogeneous environment with spatial variation in the optimal phenotype, resulting in the evolution of spatially differentiated genotypes that exhibit fitness tradeoffs when transplanted between environments (Kawecki and Ebert 2004; Savolainen *et al.* 2013). As it depends upon the maintenance of genetic polymorphism among populations, local adaptation evolves when some kind of constraint prevents a single genotype from having highest average fitness overall (*i.e.*, limited phenotypic plasticity). For example, in conifers, individuals that invest resources in defenses such as anti-freeze proteins necessarily have less resources available for growth; individuals that time their autumnal growth cessation too late are susceptible to frost damage, while those that cease growing early sacrifice productivity (Howe *et al*., 2003). Local adaptation, therefore, arises because cold environments tend to favor genotypes that increase frost tolerance or early growth cessation, whereas these genotypes are selected against in warm environments.

Local adaptation also fundamentally depends upon the tension between the strength of spatially divergent natural selection, which drives allele frequency divergence, and migration, which counteracts this divergence. Using a continent-island model, Haldane (1930) and Wright (1931) showed that an allele adapted to an island population would be lost if the rate of migration of a maladapted allele (*m*) from a continental population exceeds the strength of selection favoring the local allele (*s*). A range of other models show similar behavior, where “migration swamping” and loss of polymorphism will occur if migration is strong relative to divergent natural selection (Felsenstein 1976; Lenormand 2002).

Population genetic models lead to the prediction that when local adaptation occurs with migration, the underlying architecture should be enriched for alleles of larger effect relative to global adaptation, where there is no tension between migration and selection and no swamping (D’Ennequin *et al.* 1999; Griswold 2006; Yeaman and Whitlock 2011). This might partly explain why so many examples of alleles of large effect are found in studies of local adaptation, as described above (but see Orr and Coyne 1992 for discussion of alternative explanations). Indeed, even in humans many of the variants of largest effect are found underlying local adaptations, such as diving response in the Bajau people (*PDE10A and BDKRB2*; Ilardo *et al.* 2018), altitude adaptation in the Andes and Tibet (*EPAS1*; Bigham *et al.* 2010; Yi *et al.* 2010), and lactase digestion (*LCT*; Tishkoff *et al.* 2007). Linkage disequilibrium (LD) can also be much more important in local adaptation, as multiple tightly linked alleles tend to be inherited together, and can therefore function as if they were a single larger locus from the perspective of migration-selection balance. As the rate of recombination is a critical factor affecting LD, recombination rate tends to play a much more important role in models of local adaptation than in models of global adaptation.

This paper aims to review the predictions from theoretical models of global *vs* local adaptation and highlight some of the similarities and differences in the patterns we might expect as we scan the genome for their signatures. My review of the literature is necessarily limited to representative models that illustrate particular points and should not be taken as an exhaustive summary of the literature. My broad aim is to highlight how this theory can be usefully deployed to interpret why results from different kinds of genome studies may differ, and ultimately, to use the results of such studies to learn more about how evolution works. But first, I will begin by reviewing the concept of genotypic redundancy, which can help relate the predictions of population and quantitative genetic models.

## Genotypic redundancy: a unifying concept in population and quantitative genetics

Most, but not all, phenotypic variance depends on many loci (Orr and Coyne 1992; Johnson and Barton 2005). The standard population genetic approach of ascribing a selection coefficient to an individual locus yields tractable models but does not always extend easily to polygenic traits. If a polygenic trait is under stabilizing selection favoring some intermediate phenotype, this results in extensive epistasis for fitness, and allele frequency change at individual loci cannot be easily modeled using the population genetic approach. For example, if individual mutations have a haploid effect size of ±0.5 on a phenotype and the optimum phenotype is *Z*_opt_ = 0, then a population fixed for +,+,−,− at four diploid loci (*Z*** **=** **0) would experience deleterious selection on a new + mutation at locus 4, whereas a population fixed at −,−,−,− (*Z* = −4) would experience positive selection on the same mutation. By contrast, the classical quantitative genetic approach can be readily used to study the effect of selection on the trait mean, variance, and higher moments (Falconer and Mackay 1996), but such models do not make explicit predictions about underlying allele frequency change, and so are not as useful for studying the underlying genetic architecture.

An intermediate approach is to model selection on a phenotype determined by many loci and track how this drives the evolution of individual alleles, which experience selection through their effects on the phenotype. This polygenic approach to modeling can be deployed to arrive at analytical predictions in some special cases (*e.g*., Barton and Turelli, 1987; Jain and Stephan 2015; Höllinger *et al.* 2019), but because full models to track change at many loci can be complex, it is often better suited to numerical or individual-based simulation. With this approach, the genotypic redundancy of the trait is a critical parameter, which is determined by the relationship between the number of loci affecting a trait (*n*), the average allele effect size (α¯), and the distance to the phenotypic optimum (*D*). If *n*α¯ = *D* then there is no genotypic redundancy, so in order to reach the phenotypic optimum, alleles would have to fix at all relevant loci. In modeling terms, with no redundancy a polygenic model can be reduced to a population genetic model where each locus experiences selection in direct proportion to its additive effect on the phenotype. If *n*α¯ ≫ *D* then there is genotypic redundancy, and there are many more loci that can mutate to favorable alleles than necessary to reach the phenotypic optimum. With redundancy, the effect of selection on any allele is contingent on the genetic background, so a population genetic model would require representation of extensive epistasis for fitness to make predictions about genetic architecture. Models of multi-locus adaptation that use individual selection coefficients to represent directional or divergent natural selection implicitly assume no redundancy (*e.g*., Barton 1983; Gillespie 2004; Feder and Nosil 2010; Flaxman *et al.* 2013), while those that model selection on a phenotype under stabilizing selection implicitly assume high redundancy (*e.g*., Barton 1989; Orr 1998; Barton 1999; Le Corre and Kremer 2003; Guillaume 2011). Redundancy has been modeled explicitly in a range of theoretical approaches (Cohan 1984; Goldstein and Holsinger 1992, Turelli and Barton, 1994; Phillips 1996), and has more recently been considered as a parameter of interest in studying adaptation (Yeaman 2015; Höllinger *et al.* 2019; Láruson *et al.* 2020).

Genotypic redundancy affects a wide range of evolutionary outcomes. Most simply, if redundancy is limited then there will be high repeatability of the loci that drive adaptation among independent bouts of evolution (Yeaman 2015; Yeaman *et al.* 2018; Höllinger *et al.* 2019). However, if there are multiple bouts of adaptation from the same pool of standing variation, high repeatability could be observed even for a trait with high genotypic redundancy if the redundancy in the currently segregating alleles is low. Thus, it can be helpful to distinguish between segregating redundancy (due to alleles currently present in a population) and genotypic redundancy (due to the total mutational target that could potentially contribute; Láruson *et al.* 2020). When many different genotypes can yield the same phenotype, redundancy allows for competition among architectures, which can take on particular importance when the linkage relationships among alleles have substantial fitness consequences, as will be discussed further below. Finally, as the phenotypic distance to the optimum places a limit on the number of loci that can contribute to a trait under a scenario of no redundancy (as *n*α¯ =*D*), this implies a smaller number of loci than under high redundancy (assuming α¯ is held constant). Given that standing variation increases with the genome-wide mutation rate for a trait under mutation-selection balance (Lande 1975; Turelli 1984), which increases with the number of loci, we would therefore expect traits with high redundancy to have higher standing variation and evolvability (Yeaman 2015; Höllinger *et al.* 2019). Much of the theoretical work discussed below is based on single- or two-locus population genetic models, which provide clear predictions for polygenic adaptation with no redundancy. These models should also approximate the relative importance of evolutionary processes for traits with higher redundancy, but in some cases redundancy dramatically alters the expectation from population genetic models.

## Evolutionary process

### When does selection dominate the dynamics?

Selection causes deterministic forcing of allele frequency in the direction of higher fitness, migration homogenizes spatial differences in frequency among populations, and genetic drift adds stochastic noise to these processes. With global adaptation, the direction of selection is homogeneous across the species range, so there is no tension between migration and selection. Thus, the effect of selection on allele frequency is proportional to the selection coefficient (*s*), and if there is spatial structure, migration (*m*) mainly affects the rate of spread of a beneficial allele through the population (Fisher 1937; Ralph and Coop 2015a), with relatively little effect on the probability of fixation (Whitlock 2003, as described below).

With local adaptation, the direction of selection varies across environments, so migration opposes the divergence in allele frequency driven by selection. This dynamic is most simply captured by the continent-island model of Haldane (1930) and Wright (1931) described above, but can be extended to more complex cases like an environmental gradient, where the “characteristic length” describes the minimum spatial distance for a given change in environment to result in conditions where the effect of divergent selection outweighs migration (Slatkin 1973, 1978; see Felsenstein 1976, Bürger 2014 for other models). In a two-patch model, the tension between spatially divergent natural selection and migration can be approximated by the diversification coefficient *(***δ**), which represents their net effect on allele frequency change (Yeaman and Otto 2011; see Appendix A for more details). When **δ** > 0, the divergent forcing of allele frequencies by selection outweighs the homogenizing by migration, with the reverse for **δ** < 0. The magnitude of **δ** has a deterministic effect on allele frequency change analogous to the selection coefficient in a single-population model of directional selection (Yeaman and Otto 2011), and I will use **δ** as a shorthand for the net effect of the interplay between migration and selection on a single locus.

With either global or local adaptation, genetic drift sets an ultimate boundary on the efficiency of natural selection. If the deterministic forcing of allele frequencies is small relative to the stochastic noise introduced by genetic drift, alleles will behave as if they were neutral (Kimura 1962, 1968; Ohta 1973). With global adaptation, selection drives persistent increase in the beneficial allele when *s*** **>** **1/(4 *N_e_*) (Wright 1931; Crow and Kimura 1970), where *N_e_* is the effective population size. Similarly, with local adaptation, selection will tend to maintain a locally adapted allele when **δ** > 1/(4 *N_e_*), despite the homogenizing effect of migration and stochasticity due to drift (Yeaman and Otto 2011; for simplicity, most cases below will be discussed in terms of the sign of **δ** but it should be remembered that drift is also important). It is worth noting that the distinction between global and local adaptation becomes blurred when environments are heterogeneous and migration rates are high enough that a generalist genotype outperforms locally adapted specialist ones (as this resembles the outcome of global adaptation).

Extending the above dynamics to multilocus models, the effect of selection on a phenotype is partitioned among alleles according to their effect sizes. Even when selection on the phenotype is strong relative to migration rate, if individual alleles have small effects, then selection can be weaker than migration at the allelic level *(i.e.*, **δ** < 0; Yeaman and Whitlock 2011; Yeaman 2015). If there is no genotypic redundancy, then dynamics can be captured by extension from simple population genetic models, but if there are many different genotypes that yield the same phenotype, the net effect of selection on individual alleles will be reduced and will depend upon the genetic background. Thus, the amount of genotypic redundancy can have an important impact on how genetic architecture evolves, as a weakening of the net effect of selection on individual alleles with increased redundancy can shift migration-selection balance from **δ** > 0 to **δ** < 0.

### Fitness effects of LD and recombination

Deterministic changes in allele frequency driven by selection can be modified by linkage among loci (Hill and Robertson 1966; Lenormand and Otto 2000; Otto 2009). If two linked alleles are selected in the same direction then the effect is amplified by linkage, whereas if they are selected in opposite directions there is interference. While linkage has no particular effect on fitness within any given generation, this effect accrues to lineages over multiple generations because it maintains association among alleles (Felsenstein 1965). Thus, the combined fitness of the linked arrangement is maintained, which modifies the deterministic forcing of allele frequencies relative to what would otherwise occur under random assortment. Interference among linked alleles is commonly known as the Hill-Robertson effect (Hill and Robertson 1966; Otto 2009), and has been discussed extensively for its importance on the evolution of sex (Kimura 1956; Nei 1967; Otto and Barton 1997) and effects on adaptation (Lenormand and Otto 2000; Otto 2009).

With local adaptation, if selection is strong relative to migration and drift [**δ** > 1/(4 *N_e_*)], evolution favors alleles with larger effects, as described above. Tight physical linkage can provide another way for multiple alleles of small effect to act like one allele of large effect, and so architectures where the allelic effects on phenotype are “concentrated” in a small region of the genome tend to be favored (Bürger and Akerman 2011; Yeaman and Whitlock 2011). Some simple rules of thumb about the importance of linkage in local adaptation can be derived from a two-locus continent-island model: if a locally adapted allele established in an island (with selection coefficient = *s_b_*; assume *s_b_* > *m*) is linked to another locus experiencing weaker selection (coefficient = *s_a_*) with recombination rate *r* between them, selection will deterministically favor a new locally adapted mutation at the linked locus when *r* < *s_a_s_b_*/*m* (Yeaman *et al.* 2016). This shows that locally adapted alleles with *s_a_* ≪ *m (i.e.*, **δ** < 0) can still be deterministically favored if linkage is sufficiently tight. If we assume *s_a_* **_*∼*_** *m*, this reduces to Barton’s (2000) rule of thumb that selection will exert an effect at linked sites when *r* < *s_b_*. Similar thresholds can be derived for more complicated models (*e.g*., Akerman and Bürger, 2014); for simplicity, I will use δ* to represent the net effect of selection, migration, and linkage to other alleles on the deterministic forcing of allele frequencies at a focal locus (such that when δ* > 0, divergently selected alleles at the focal locus tend to be maintained, even if **δ** < 0). The difference between δ* and **δ** can then approximate the fitness advantage due to linkage, with selection operating efficiently when this difference is large relative to genetic drift. It is worth noting that the interaction between evolutionary processes described here also applies to some forms of balancing selection, such as negative frequency-dependent selection (Kopp and Hermisson 2006; van Doorn and Dieckmann 2006; Schneider 2007).

## Evolution of genetic architecture

**Figure 1:**
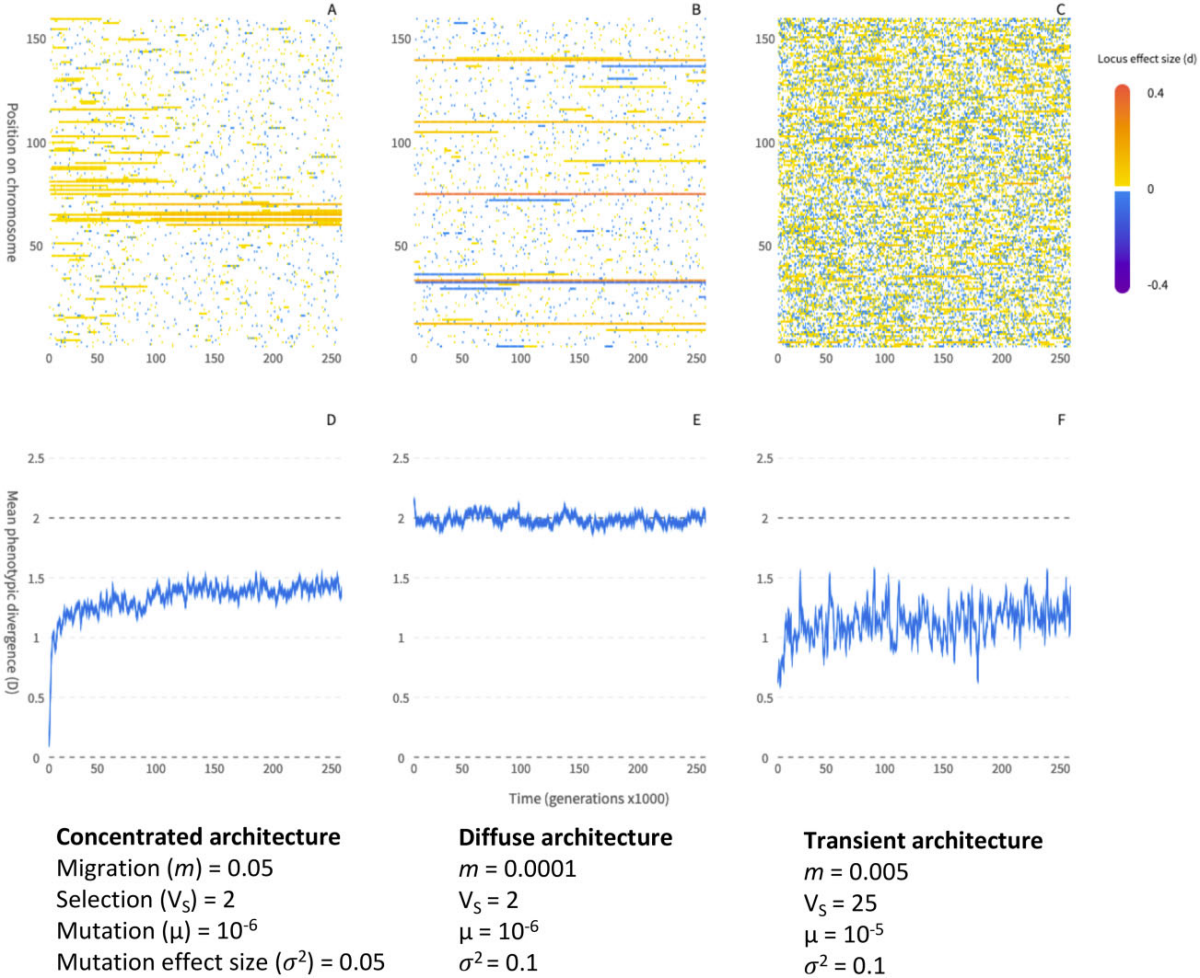
Local adaptation can occur with very different underlying genetic architecture, depending on the balance between migration and selection, allele effect size, drift, mutation rate, and genotypic redundancy. Panel (A) shows a concentrated architecture, panel B shows a stable diffuse architecture, and panel C shows a transient architecture. Panels (D–F) show the mean phenotypic divergence (*D*) between two simulated populations experiencing stabilizing selection toward local optima of ±1 (such that optimal local adaptation occurs when *D *=* *2); panels A–C show the contribution of each locus to phenotypic differentiation (*d*) for 160 equally spaced loci along a simulated chromosome with an even rate of recombination. Simulations differ according to the parameters shown below each scenario, where *V_S_* is the width of the Gaussian fitness function for stabilizing selection (lower values result in stronger selection), the mutation rate (µ) is per locus, and σ^2^ is the width of the Gaussian function for mutation effect sizes (see Appendix B for simulation details). The concentrated architecture in Panel A evolves mainly through competition among alleles with different linkage relationships. In panel B, migration is low and so there is little advantage for clustering of linked alleles and little architecture evolution. In panel C, individual alleles are often large enough to resist swamping (δ > 0) but the high redundancy and mutation rate result in a large number of alleles segregating at any given time, resulting in rapid turnover in the evolved architecture.

It is clear from the above that local adaptation with migration will tend to favor concentrated architectures enriched for alleles of larger effect, clustered into a smaller number of genomic regions, relative to global adaptation. This difference will be most pronounced at intermediate migration rates—high enough to yield an advantage for linkage but not so high as to prevent the stable maintenance of differences in allele frequency. At low migration rates, local adaptation will more closely resemble global adaptation (Figure 1A  *vs*  Figure 1B). Concentrated architectures can evolve due to differences between linked *vs* unlinked alleles in their establishment probability or persistence time once established, or through competition among established alleles and replacement of loosely linked architectures by more concentrated ones. I now review the conditions required for each of these mechanisms to lead to the evolution of concentrated architectures, and discuss the conditions when local adaptation may evolve via other kinds of underlying architecture.

### Clustering via differential establishment probability

**Figure 2:**
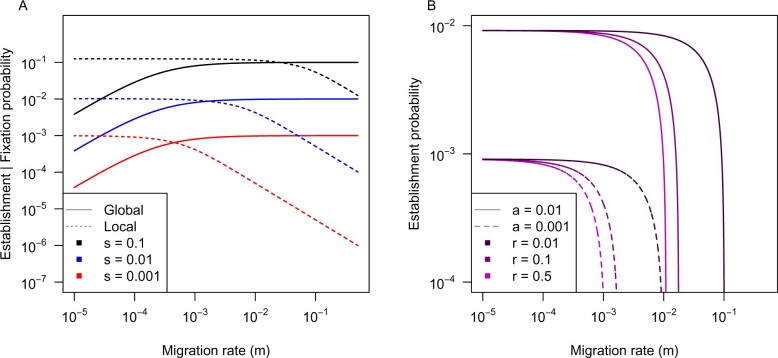
Comparison of the probability of a new mutation rising to fixation under global adaptation *vs* establishment under local adaptation (A) and the effect of linkage with local adaptation (B). Under global adaptation with spatial structure a decrease in fixation probability with decreasing migration occurs over approximately the same migration rates regardless of the strength of selection (*s*; A). By contrast, with local adaptation a reduction in establishment probability with increasing migration occurs over lower migration rates for more weakly selected mutations, but over higher migration rates for more strongly selected ones (A). Linkage to an existing locally adapted polymorphism dramatically increases the establishment probability of new mutations (B), but this is most pronounced within a narrow zone of migration rates, which shifts with the strength of selection on the new mutation (*a*). Panel A contrasts the global adaptation model of Whitlock (2003) with the two-population local adaptation approximation of Yeaman and Otto (2011; Equation 3), but splicing δ into 2 *s N_e_*/*N*_tot_ (instead of Kimura’s equation) and assuming *N_e_* = *N*_tot_ = 1000. Panel B shows the continent-island splicing approximation of Yeaman *et al.* (2016; Equation 7) with strength of selection of *b *=* *0.1 on the established allele, strength of selection of *a* on the new mutation, and recombination rate *r* between loci.

Under global adaptation without spatial structure, fixation of a new favorable mutation is well described by Kimura’s equation (Kimura 1962). When there is spatial structure, fixation probability decreases with decreasing migration rate (1 − *F_ST_*), but this effect applies irrespective of the selection coefficient of the mutation (Barton 1993; Whitlock 2003; Figure 2A). Thus, structure should not dramatically affect the genetic architecture of global adaptation. For a bout of global adaptation toward a stable optimum, Orr (1998) showed that the mutations contributing to adaptation would tend to have an approximately exponential distribution of effect sizes. If two universally beneficial mutations occur at different loci in the same population at the same time, selective interference (*i.e.*, Hill-Robertson effect; Hill and Robertson 1966) will reduce their probability and rate of fixation (Otto and Barton 1997; Roze and Barton 2006). Such interference is less severe with high recombination between the mutations, so if anything, global adaptation will favor minimal clustering of new mutations on chromosomes (Otto 2009; Höllinger *et al*. 2019). Given that such effects only operate while alleles segregate, mutations that fixed previously in an adaptive walk do not affect new mutations, so the overall effect of selective interference favoring establishment of mutations with different linkage relationships is very weak (Otto 2009).

**Table 1:** Definition of symbols

Symbol	Definition
*S*	Selection coefficient acting on an allele
*M*	Migration rate
*N_e_*	Effective population size
*R*	Recombination rate
*Z*	Individual trait value
*Z*_opt_	Optimal trait value
*N*	Number of loci that can mutate to yield variation in a trait
*D*	Difference in *Z*_opt_ between populations
*D*	Contribution of a locus to phenotypic divergence
Α	Allele effect size
α-	Average allele effect size
δ	Diversification coefficient, indicating the net effect of the deterministic balance between divergent selection and migration
δ*	Modified diversification coefficient after accounting for the effect of linkage to other locally adapted alleles

For single-locus models of local adaptation, Kimura’s equation (1962) also provides a good approximation for the probability of establishment when **δ** is substituted for *s* (Yeaman and Otto 2011), with more exact models providing similar predictions (Tomasini and Peischl 2018; Sakamoto and Innan 2019). Because weakly selected locally adapted mutations are susceptible to swamping, their establishment probability is more strongly reduced by migration than strongly selected mutations (Figure 2A). If a new mutation occurs on a background with an established allele selected in the same direction, then tight linkage between them is beneficial and the increased probability of establishment can be approximated by substituting δ* for *s* in Kimura’s equation (Yeaman and Whitlock 2011; Yeaman *et al.* 2016; Figure 2B) and can also be derived using other more precise approaches (Aeschbacher and Bürger 2014; Yeaman *et al.* 2016).

Unlike in global adaptation, locally adapted polymorphisms are maintained for a long time under migration-selection balance, so this mechanism can potentially influence the evolution of genetic architecture over longer periods of time. The increase in establishment probability due to linkage is most pronounced within relatively narrow ranges of migration rate, and these ranges shift with the strength of allelic selection (Figure 2B). Thus, for a given migration rate, only mutations falling within a narrow range of effect sizes (*s*) will experience a very large effect of linkage on their probability of establishment. If the genome has many chromosomes and recombination rate is relatively homogeneous, the modest increase in establishment probability in linked regions may be outweighed by the larger number of mutations occurring in unlinked regions, in which case this mechanism is unlikely to yield strong signatures of clustered alleles (Yeaman 2013; Yeaman *et al.* 2016). However, if inversions or other features reduce recombination rate over larger chromosomal regions, the advantage due to linkage could dramatically increase the potential for clustering under this mechanism (Yeaman *et al.* 2016). Because the establishment probability of a new mutation is proportional to the strength of selection, differences in establishment probability are less likely to drive architecture evolution when there is high segregating redundancy (as this will result in competition among architectures).

### Clustering via competition among architectures

When there is genotypic redundancy, combinations of alleles yielding the same phenotype but differing in their linkage relationships would have equal fitness within a generation, but increased/reduced fitness averaged over subsequent generations due to the effect of linkage, as described above. Under global adaptation, selection favoring modifiers of recombination among loci tends to be weak and only operates while variation persists (Maynard Smith 1977; Lenormand and Otto 2000; Otto 2009), so competition among architectures with the same phenotype but different linkage relationships tends to be weak. In this case, evolutionary dynamics are mainly governed by the interplay between selection, drift, and mutation rate at any redundant loci (Höllinger *et al.* 2019) and selection does not tend to favor the evolution of clustering of causal loci (Yeaman 2013).

Under local adaptation, because of the general advantage for tighter linkage and/or larger allele effect size, competition will favor the evolution of concentrated genetic architectures with larger and more tightly linked alleles, clustered in a smaller number of regions of the genome (D’Ennequin *et al.* 1999; Yeaman and Whitlock 2011). New mutations yielding a more concentrated architecture will then invade and outcompete less concentrated alleles with phenotypically redundant effects (as shown in Figure 1A). The advantage of a more concentrated architecture over one with unlinked alleles of the same size is approximately proportional to δ*- **δ**, which increases with migration rate and strength of selection on the phenotype (as long as δ* > 0), and also depends upon the difference in effect size or linkage relationship between the competing architectures (Yeaman and Whitlock 2011). As such, the strength of selection on different architectures with the same phenotype tends to be much weaker than the strength of selection on the individual alleles (Bürger and Akerman 2011; Yeaman and Whitlock 2011; Aeschbacher and Bürger 2014). Competition among allelic architectures therefore tends to reshape adaptation very gradually, depending also on the mutation rate and amount of redundancy, and would require prolonged periods where heterogeneous environments persistently favored the maintenance of local adaptation (Yeaman and Whitlock 2011).

It is also possible for competition to occur among “genomic architectures” that have the same alleles but differ in the rate of recombination between these alleles, due to some change in the underlying genome organization or meiotic behavior of the chromosome. This can occur due to a modifier of recombination such as the loss of a particular motif guiding meiotic crossing-over (*e.g*., *PRDM9*; Paigen and Petkov 2018) or the fixation of a chromosomal rearrangement that moves loci into tight physical linkage (Yeaman 2013; Guerrero and Kirkpatrick 2014). Similarly, if a chromosomal inversion occurs that captures multiple locally adapted alleles, recombination will be suppressed between the inverted and un-inverted arrangements, thereby favoring the spread of the inversion in populations where its alleles are favored (Kirkpatrick and Barton 2006; Bürger and Akerman 2011; Charlesworth and Barton 2018). While these different mechanisms reduce recombination in different ways, they all have the net effect that more LD can be maintained between locally adapted alleles, which confers higher fitness on average. Unlike competition among allelic architectures, competition among genomic architectures does not require genotypic redundancy and likely progresses more rapidly if redundancy is low, as the strength of this effect scales positively with the strength of selection on the individual locally adapted loci (Bürger and Akerman 2011; Yeaman and Whitlock 2011), although this has not been explicitly studied. The most important difference between competition between alleles *vs* rearrangements is that the latter lead to durable changes in the underlying genome architecture that would persist through population bottlenecks causing loss of polymorphism (and loss of a concentrated allelic architecture; Yeaman 2013).

### Clustering via differential maintenance of selected polymorphisms

If local adaptation occurs along with increasing migration rates, which can occur during secondary contact and hybridization among previously separated populations, then alleles with lower **δ** or δ* may be lost more readily, leading to a more concentrated architecture (Rafajlović *et al.* 2016; Yeaman *et al.* 2016). If there is high segregating redundancy, loss of the less concentrated alleles can simply be a part of competition among architectures, but this mechanism can still operate if there is no redundancy and no scope for competition.

### Adaptation with a transient underlying architecture

The typical conception of adaptation implies a temporally stable change in genotype: a new mutation invades and replaces an old one. However, with local adaptation, a consistent difference in mean phenotype can be maintained even with constant turnover in the underlying alleles that contribute to divergence. If individual alleles experience weak divergent selection relative to migration *(***δ**<0), swamping will tend to prevent long-term maintenance of polymorphism (Felsenstein 1976; Bürger 2014). Despite this apparent population genetic limit to local adaptation, phenotypic divergence can still be maintained by selection driving small differences in allele frequency at many loci (Latta 1998; Le Corre and Kremer 2003; Yeaman 2015). Because the effect of selection on any given allele is weak, these differences tend to be homogenized by migration, so the underlying divergence at individual loci is transient. As quantitative genetic models show that divergence scales linearly with standing variation (Hendry *et al.* 2001), this mode of local adaptation depends critically on the maintenance of standing variation. When migration is strong relative to selection on individual alleles *(***δ** < 0), standing variation is maintained mainly by mutation, so phenotypic divergence by this mechanism is most pronounced when mutation rate and genotypic redundancy are high (Yeaman 2015).

The architecture of local adaptation can also become transient, with turnover in the alleles that contribute to divergence even when individual alleles are resistant to swamping *(***δ** > 0), if segregating redundancy is high (Yeaman 2015). This will occur when mutation rate is high and there is substantial underlying genotypic redundancy, such that many different combinations of alleles with high fitness are present in the population. This leads to rapid turnover in the alleles that contribute to local adaptation (Figure 1C), presumably because the advantage of one architecture over another is small relative to drift, although this has not been studied extensively.

Analogous results are found in models of adaptation to a new globally uniform environment. When redundancy is high, there are many potential ways that adaptation can achieve a given change in phenotype, and response to selection will tend to involve many small shifts in allele frequency (Jain and Stephen 2015, 2017; Höllinger *et al.* 2019). Prolonged stabilizing selection after the optimum is reached will then result in turnover of the alleles that contribute to adaptation (Barton 1989). When redundancy is low there are fewer viable ways to achieve a new adaptive phenotype, individual alleles will need to experience larger changes in frequency to achieve the new optimum, and there will be less chance for turnover once the optimum is reached. Depending upon the distance between the old and new optimum, the number of loci, allele effect sizes, and amount of redundancy, adaptation to a global optimum can therefore proceed by many small shifts or a few large allele frequency sweeps. Höllinger *et al.* (2019) showed that a critical parameter in determining whether shifts or sweeps will predominate is the total population mutation rate at all redundant loci, which is analogous to the shift in regime from stable to transient underlying architecture that occurs with increasing mutation rate and redundancy in models of local adaptation (Yeaman 2015).

### Reduced concentration of genetic architecture under temporal heterogeneity

Adding temporal variation in the phenotypic optimum to models of local adaptation can dramatically affect their predictions about the evolution of concentrated architectures. When the locally optimal phenotype changes, it becomes advantageous to break up associations between alleles to generate new combinations and new phenotypes that better match the new environment, which favors higher recombination (Kondrashov and Yampolsky 1996; Bürger and Gimelfarb 2002; Otto 2009). In a model where rearrangements allow for the evolution of genome organization, spatial heterogeneity led to clustering, but when temporal heterogeneity was added as well, a hybrid architecture was observed where some loci were clustered (to deal with space) and some were dispersed (to deal with time; Yeaman 2013). Whereas spatial heterogeneity and local adaptation tend to favor clustered architectures, temporal heterogeneity tends to favor dispersed ones. Temporal heterogeneity can also increase the maintenance of genetic variation (Bürger and Gimelfarb 2002; Gulisija and Kim 2015; Wittmann *et al.* 2017), which might change the architecture of local adaptation from a stable regime to a transient one, if many redundant genotypes are present in the population at once. Given the complexity involved and limited work on this subject, the combined effect of spatial and temporal heterogeneity on genetic architecture remains an important area for future research.

### Conditional neutrality

There are important differences in the predictions about genetic architecture of local adaptation if some mutations have fitness effects that are neutral in one environment and beneficial or deleterious in the other, termed conditional neutrality (Fry 1996; Kawecki 1997; Anderson *et al.* 2013). Under this scenario, although one allele is fitter on average and will therefore eventually fix, a signature of local adaptation (*i.e.*, fitness tradeoffs in a reciprocal transplant experiment; Kawecki and Ebert 2004) can be maintained if recurrent mutation results in alleles that are conditionally neutral in one environment or the other segregating at multiple loci. Whereas divergent selection results in a tension with migration that favors concentrated architectures, there is no such tension with mutations that are conditionally neutral. Thus, predictions about genetic architecture for conditionally beneficial mutations are similar to those for global adaptation, while the load induced by conditionally deleterious mutations (Mee and Yeaman 2019) has more in common with conventional genetic load (Bürger 2000).

An interesting problem emerges if conditionally deleterious mutations occur along with divergently selected ones. Suppose that local adaptation results in the emergence of a concentrated architecture with a divergently selected allele of large effect (or a cluster of several small ones). This architecture generates substantial LD and reduces the effective migration rate in its flanking regions (the “barrier effect”; Barton and Bengtsson 1986). If conditionally deleterious mutations are also occurring randomly throughout the genome, they would be expected to accumulate faster in these flanking regions, where the effective migration rate is lower (as the expected load for conditionally deleterious mutations, *s*µ*n*/*m*, increases with reduced migration; Mee and Yeaman 2019). In the event of a change in environment, this conditionally deleterious load would be revealed in addition to any now-maladaptive consequences of the previous local adaptation due to the concentrated architecture. Thus, the average benefit of a concentrated architecture may be partially offset by the accumulation of conditionally deleterious load in its flanking regions, especially if environments also fluctuate over time. While the fitness advantage of concentrated architectures can potentially reshape the genome through chromosomal rearrangement (Yeaman 2013), it is unclear if conditionally deleterious load might counterbalance this evolutionary pressure, so further theoretical work is required.

### The effect of spatial structure

Spatial structure is inherent to models of local adaptation, but it is unclear how readily predictions from simple two-patch models generalize to more realistic scenarios such as clines or patchy two-dimensional landscapes. While focused exploration is warranted, it seems likely that the qualitative differences in architecture described here (*e.g*., Figure 1) will also extend to these more realistic scenarios. One of the most important consequences of spatial structure is the potential for adaptation to evolve semi-independently in different areas of a species range. When this occurs, we may see different architectures of adaptation in different regions, especially with high genotypic redundancy. This has been explored for global adaptation in the interplay between mutation and migration rate: if the population mutation rate at a single locus is high, then different parts of a species range may independently evolve the same mutation, whereas if migration rate is high then a single mutation is more likely to spread to all regions (Ralph and Coop 2015a). High mutation relative to migration under global adaptation can therefore result in a pattern of spatial differentiation in alleles that resembles local adaptation. This logic can be extended to high redundancy, whereby if mutation rate is high across multiple loci, repeatability of the genetic basis of adaptation will be low across the species range. Similar models can be constructed for local adaptation—if there are repeated environmental gradients across a species range then migration among the gradients will affect whether similar or different architectures of adaptation evolve along each gradient (Ralph and Coop 2015b). Given the importance of mutation, these considerations may be particularly relevant for traits with a high net mutation rate, such as microsatellites driving limb and skull morphology in dogs (Fondon and Garner 2004) or a fragile DNA site that has yielded repeated deletions causing loss of pelvic hindfins in stickleback (Xie *et al.* 2019). In general, traits that evolve via loss-of-function mutations may experience higher average rates of new mutation (as there are usually more ways to break a function than improve it), and indeed loss-of-function mutations are often found contributing to adaptation (Behe 2010; Xu and Guo 2020). As global adaptation in a trait with a high mutation rate can yield spatial structuring in allele frequencies that resembles local adaptation (Booker *et al.* 2021), it is important to consider the effect of mutation rate on the evolution of genetic architecture.

### Summary: how will genetic architecture evolve?

All else being equal, we expect the genetic architecture of local adaptation to involve fewer, larger, and more tightly linked alleles than global adaptation (Orr 1998; Griswold 2006; Yeaman and Whitlock 2011). However, as the selection pressures involved in architecture evolution are weak in comparison to those acting directly on alleles, there may be little realized difference between global and local adaptation in nature, where drift may limit the efficiency of selection. Concentrated architectures will evolve most rapidly under the following conditions: (1) migration rate is high, but still below the swamping limit for a substantial fraction of alleles *(i.e.*, some alleles have **δ** > 0), as this maximizes the advantage of linkage for alleles of smaller effect that would otherwise experience swamping *(i.e.*, those with **δ** < 0); (2) population size (*N*) is large, as architecture evolution is limited by the availability of standing variation or the rate of new mutations at redundant sites or the occurrence of structural rearrangements, all of which will increase with *N*, as does the efficiency of selection; (3) the spatially heterogeneous environment presents a strong and temporally consistent divergent selection pressure.

The effect of genotypic redundancy on the evolution of architecture is complex: on the one hand, without some redundancy there will be little scope for competition among alleles and the only way to evolve a concentrated architecture is to rearrange the underlying loci. On the other hand, if redundancy is very high, then individual alleles likely experience weaker selection (limiting the advantage of linkage) and in extreme cases, there may be so much variation present that architectures become transient due to rapid turn-over of alleles (*e.g*., Figure 1C). Concentrated architectures would likely evolve most rapidly under a scenario with mixed redundancy, where there are some genes that are particularly well-suited to contributing to adaptation via alleles of large effect (with low redundancy) and a large number of genes with redundant effects on the phenotype that tend to yield mutations of smaller effect. Under this scenario, alleles of large effect would readily establish and contribute to local adaptation, with subsequent fine-tuning of the phenotype occurring through preferential establishment/competition favoring alleles of smaller effect at closely linked sites. Given our limited knowledge about the extent of genotypic redundancy and how it may also be shaped by evolution (Láruson *et al.* 2020), it is unclear whether concentrated architectures will commonly be seen in nature, and whether some kinds of traits or environments will be more likely to evolve via one kind of architecture or another. Further theoretical work studying how evolution shapes redundancy itself is needed.

## Empirical evidence and future directions

The theory reviewed above makes some clear predictions about the evolution of genetic architecture, but are such predictions actually borne out in nature? The three-spine stickleback seems to provide one of the most striking examples of a concentrated genetic architecture underlying local adaptation. Early fine-scale mapping of the genetic basis of marine-freshwater divergence found an allele at the *Eda* locus driving a large proportion of variation in armor plating (Colosimo *et al.* 2005), and subsequent studies have identified other causal variants in tight linkage with the *Eda* allele (Howes *et al.* 2017; Archambeault *et al.* 2020). Given selection on the *Eda* haplotype of *s* **∼** 0.5 (Schluter *et al.* 2021), other freshwater-adapted alleles would experience an advantage if clustered within 50 cM of *Eda* (based on the *r* < *s_a_s_b_*/*m* rule of thumb), which in practice means that a concentrated architecture could extend through most of the chromosome where *Eda* resides. Indeed, genome-wide divergence between marine and freshwater populations is elevated in large “genomic islands” around *Eda* and also in a few other regions of the genome (Hohenlohe *et al.* 2010; Jones *et al.* 2012), and these islands tend to be enriched for Quantitative Trait Loci (QTL) affecting multiple locally adapted traits (Peichel and Marques 2017). One of these regions on chromosome XXI is enriched for QTL affecting tooth, jaw, and vertebrae phenotypes (Miller *et al.* 2014), with two closely linked causal loci identified within the region (*Bmp6 and Tfap2a*; Cleves *et al.* 2014; Erickson *et al.* 2018). It is unclear how many other undetected causal loci may be involved in these genomic islands, but the evidence seems consistent with some advantage for clustering playing a role in the architecture of local adaptation. Stickleback have an ecology that may be particularly suitable for the evolution of a concentrated architecture, as freshwater-adapted alleles persist as standing variation in marine populations (Schluter and Conte 2009; Nelson and Cresko 2018). Over millions of years, repeated bouts of colonization of freshwater environments from this standing variation would therefore provide ample opportunity for gradual evolution of increasingly concentrated architectures through several of the mechanisms discussed above.

Beyond the stickleback, there are now numerous examples of alleles of large effect driving local adaptation (see Introduction), inversions are commonly associated with local adaptation (Wellenreuther and Bernatchez 2018), and clustered architectures have been found where a QTL affecting multiple traits can be decomposed using fine-scale linkage mapping to reveal a number of tightly linked variants each affecting a different trait or subset of traits (Christians and Senger 2007; Hermann *et al.* 2013). In a fascinating study of divergent adaptation in yeast, a comparison of lab *vs* vineyard strains using CRISPR-based assays found that causal variants with fitness effects in the same direction tended to be clustered together on chromosomes (Sharon *et al.* 2018). These examples certainly seem like concentrated architectures consistent with the predictions described above—but did they evolve because of the advantage of linkage under migration-selection balance? And if so, did they evolve only through differential success of larger/clustered alleles or did adaptation also reshape the architecture of the genome through rearrangement? It is also critical to consider other explanations for why alleles of small *vs* large effect may respond differently to selection regardless of migration rate, such as interactions between the effect size, degree of pleiotropy, and strength of selection (Orr and Coyne 1992; Crow 1957).

To explicitly test whether some concentration of architecture evolves because of local adaptation, it is necessary to deploy a comparative or experimental approach. This could be done by contrasting natural populations adapting to a similar environmental gradient under high *vs* low migration (*e.g*., Holliday *et al.* 2016), using experimental evolution where such parameters are controlled (*e.g*., Tusso *et al.* 2021), or comparing patterns more broadly across a large number of species or across space *vs* time to test the effect of some covariate of potential importance (*e.g*., migration rate, population size, and so on). For the latter approach in particular, it necessary to develop standardized statistics to enable comparisons of genome scan results across studies: what does a given Manhattan plot tell us about the number, clustering, and effect size of causal alleles? Early genome scans identified highly heterogeneous patterns of divergence in allele frequency among populations (Nosil *et al.* 2009), but it is usually unclear if these genomic islands include multiple causal alleles or a single allele of large effect with hitchhiking neutral alleles in flanking regions. Given that other evolutionary processes such as global adaptation or background selection can also potentially drive such signatures (Noor and Bennett 2009; Cruickshank and Hahn 2014; Matthey-Doret and Whitlock 2019; Booker *et al.* 2021), it will be difficult to confidently assess the genetic architecture of adaptation using genome scans alone. Complementing environmental-association or *F_ST_*-based genome scans with studies of allele frequency change over time, trait-based GWAS, or targeted crosses and fine-scale mapping could greatly improve the power to assess whether causal mutations are clustered. Where possible, targeted manipulations via approaches like CRISPR (Sharon *et al.* 2018) can provide the strongest proof of causality.

At the other end of the spectrum, given that high redundancy and mutation rates can result in transient architectures underlying local adaptation (*e.g*., Figure 1C), the failure to find concentrated architectures is a fundamentally interesting result, but only if framed in terms of the statistical power (what is the maximum effect size that could have gone undetected?). There are examples of local adaptation at the phenotypic level with no evidence for alleles of large effect or clustering (*e.g*., Ehrlich *et al.* 2020), but it is difficult to rigourously demonstrate the absence of a pattern on the massive scale of genomic data, especially with methods that do not fully sample the genome (Lowry *et al.* 2017).

As our understanding grows about how commonly concentrated architectures evolve and why (or why not), we can use this to answer more fundamental questions about adaptation: Is the set of variants that contribute to adaptation flexible or constrained? How many different ways can a species adapt to the same stress? If we see the same loci contributing to independent bouts of adaptation repeatedly in different species, we can infer that the underlying genotype-phenotype-fitness map has low redundancy (Yeaman *et al*., 2018). Such low redundancy may arise because there are only a few loci that can yield mutations that affect a phenotype under selection, or because many loci can yield mutations affecting the phenotype, but only a subset of them have the highest fitness, due to pleiotropy or other side-effects. These two explanations imply very different constraints to evolution. Given that expectations for architecture evolution can differ dramatically for global *vs* local adaptation, to understand redundancy through the lens of genetic architecture, we must interpret data in light of which kind of selection is operating. As an example, as migration swamping prevents alleles of small effect from contributing to local adaptation, then increased repeatability of adaptation might be observed at high migration rates if only a subset of loci can yield mutations of large effect *(i.e.*, **δ** > 0). This would lead to an inference of lower redundancy than would be found for a similar scenario without migration (where many alleles of small effect could also contribute), so it is important to understand the causal reasons for this difference.

Returning to the questions posed at the beginning of this study, what is the nature of trait variation in general? If GWAS tend to find many alleles of predominantly small effect underlying standing variation, is this actually indicative of the adaptive potential of the species? From a cursory look at best-studied examples of local adaptation where alleles of large effect are commonly found, we might conclude that there is little similarity between distributions of allele effect size for GWAS (*i.e.*, standing variation) *vs* causal drivers of adaptation. However, when we interpret this difference in light of the theoretical expectation that alleles of large effect should prevail under migration-selection balance, then perhaps there is less discrepancy between these observations. Alternatively, the difference may be one of process in general: if most mutations are basically deleterious on average—albeit with correlated effects on phenotypes of interest—then most standing variants would never ultimately contribute to adaptation despite contributing to quantitative genetic variation. It remains to be seen whether the alleles that contribute to standing variation in GWAS are the “stuff” of long-term adaptation. Experimental evolution studies have shown considerable redundancy in the response to selection (Barghi *et al.* 2019) and that short-term change is well described by quantitative genetic models that account for alleles of large effect (Castro *et al.* 2019). But will such short-term experiments conducted at relatively small population sizes prove to be representative of longer-term adaptation? Answering this question will require systematic comparison of the variants that contribute to adaptation *vs* standing variation, along with an accounting for how the evolutionary pressures involved in the local *vs* global regime may have shaped the observed set of adaptive variants.

## Data availability

There are no data associated with this manuscript; scripts to run SLiM3 are available at www.github.com/jcbain/descartes.

## Appendix A: Diversification coefficient

The diversification coefficient (δ) is derived for a two-patch model and represents the deterministic rate of increase in frequency of a locally favored allele when rare, due to the combined effects of migration and selection (Yeaman and Otto 2011). This δ is analogous to the selection coefficient (*s*) favoring heterozygotes in a classic deterministic one-locus model of directional selection. The full equation for δ is:

(A1) $$\delta =\frac{\psi +\sqrt{\psi^{2}-4\left(1-2m\right)\frac{w_{1,Aa}}{w_{1,AA}}\frac{w_{2,Aa}}{w_{2,A\text{A}}}}}{2}-1$$

where ψ=1-mw1,Aaw1,AA+w2,Aaw2,AA, *w_i, j_* is the relative fitness of the *j*^th^ genotype in the *i*^th^ patch, and allele *a* is the rare allele that is invading. For alleles that affect a phenotype, the fitness of the various genotypes can each be calculated based on their phenotypes (*Z_j_*), the local optimum (*Z*_opt_), and the shape of the fitness function (*e.g*., V_S_ for Gaussian selection). When applied to a polygenic trait, it must be assumed that all individuals have the same genetic background (*Z*) in order to calculate the fitness coefficients (*w_i, j_*), but this will overestimate δ if there is considerable standing variation and some phenotypes overshoot the local optima (as the average strength of selection acting on a single locus will be weaker). On the other hand, if locally adapted alleles are segregating at many other loci then Equation A1 will not account for the effect of linkage, thereby underestimating the magnitude of δ. Approximations accounting for linkage (δ*) can be derived (*e.g*., Yeaman *et al.* 2016) but this becomes complicated for more than two loci. In order for a locally adapted polymorphism to be stably maintained, δ > 0 must be satisfied for the invasion of each allele when rare (*i.e.*, solving Equation A1 twice, letting each allele be *a*).

## Appendix B: Individual-based simulations

Individual-based simulations illustrating local adaptation in Figure 1 were run using SLiM3 (Haller and Messer 2019) with two patches experiencing Gaussian stabilizing selection (with width = V_S_) toward different local optimal (±1) and migration (at rate *m*). Individuals had diploid genomes with 160 loci arranged on a single chromosome, each separated by recombination rate of *r *=* *0.00625, so that on average there is one recombination event per chromosome per generation. Each locus was modeled as a QTL experiencing recurrent mutation (at rate µ per locus) with the “last” stacking setting, whereby a new mutation at a given locus replaces the value of the previous allele. Mutation effects were additive and drawn from a Gaussian distribution with width σ^2^. Simulations were initialized with no standing variation and a population size of *N *=* *1000 individuals per population, and run for 250,000 generations, censused every 1000 generations. The contribution of each locus to phenotypic divergence among populations (*d*) was calculated as d=(∑i=12Nαi-∑j=12Nαj)/2N, where α_*i*_ and α_*j*_ are the effect sizes of the alleles present in population 1 and 2, respectively.
